# Specific Physical Ability Prediction in Youth Basketball Players According to Playing Position

**DOI:** 10.3390/ijerph19020977

**Published:** 2022-01-16

**Authors:** Jelena Ivanović, Filip Kukić, Gianpiero Greco, Nenad Koropanovski, Saša Jakovljević, Milivoj Dopsaj

**Affiliations:** 1 Serbian Institute for Sport and Sports Medicine, 72 Kneza Višeslava Street, 11030 Belgrade, Serbia; jelena.ivanovic@rzsport.gov.rs; 2 Faculty of Sport, University “Union—Nikola Tesla”, Narodnih Heroja 30/I, 11070 Belgrade, Serbia; 3 Police Sports Education Center, Abu Dhabi Police, Abu Dhabi 253, United Arab Emirates; filip.kukic@gmail.com; 4 Department of Basic Medical Sciences, Neuroscience and Sense Organs, University of Study of Bari, 70121 Bari, Italy; 5 Department of Criminalistics, University of Criminal Investigation and Police Studies, 11080 Belgrade, Serbia; nenad.koropanovski@kpu.edu.rs; 6 Faculty for Sport and Physical Education, Belgrade University, 11000 Belgrade, Serbia; sasa.jakovljevic@fsfv.bg.ac.rs (S.J.); milivoj.dopsaj@gmail.com (M.D.); 7 Institute of Sport, Tourism and Service, South Ural State University, 454080 Chelyabinsk, Russia

**Keywords:** measurement, power test, speed test, change of direction speed test, guard, forward, center

## Abstract

This study investigated the hierarchical structure of physical characteristics in elite young (i.e., U17-U19) basketball players according to playing positions. In addition, their predictive value of physical characteristics was determined for the evaluation of players’ physical preparedness. Sixty elite male basketball players performed 13 standardized specific field tests in order to assess the explosive power of lower limbs, speed, and change-of-direction speed. They were divided into three groups according to playing positions (guard [*n* = 28], forward [*n* = 22], center [*n* = 10]). The basic characteristics of the tested sample were: age = 17.36 ± 1.04 years, body height = 192.80 ± 4.49 cm, body mass = 79.83 ± 6.94 kg, and basketball experience = 9.38 ± 2.10 years for guards; age = 18.00 ± 1.00 years, body height = 201.48 ± 3.14 cm, body mass = 90.93 ± 9.85 kg, and basketball experience = 9.93 ± 2.28 years for forwards; and age = 17.60 ± 1.43 years; body height = 207.20 ± 3.29 cm, body mass = 104.00 ± 9.64 kg, and basketball experience = 9.20 ± 1.62 years for centers. For all playing positions factor analysis extracted three factors, which cumulatively explained 76.87, 88.12 and 87.63% of variance, respectively. The assessed performance measures were defined as significant (*p* < 0.001), with regression models of physical performance index (PP_INDEX_). PP_INDEX_ of guards = −6.860 + (0.932 × *t*-test) − (1.656 × Acceleration 15 m) − (0.020 × Countermovement jump); PP_INDEX_ of forwards = −3.436 − (0.046 × Countermovement jump with arm swing) − (1.295 × Acceleration 15 m) + (0.582 × Control of dribbling); PP_INDEX_ of centers = −4.126 + (0.604 × Control of dribbling) − (1.315 × Acceleration 15 m) − (0.037 × Sargent jump). A model for the evaluation of physical performance of young basketball players has been defined. In addition, this model could be used as a reference model for selection procedures, as well as to monitor the efficacy of applied training programmes within the short, medium and long-term periodization.

## 1. Introduction

Body height, muscular power, speed, and strength are all important elements of the basketball player profile. Power, speed, and change of direction speed significantly contribute to the movement efficiency of basketball players with the ball and without it, as well as in technical and tactical elements of basketball game [1,2,3]. While body height is genetically predetermined, power, speed and change of direction speed are subject to training adaptation and could be used for the assessment of players’ physical potential to overcome the challenges of a basketball game [3,4,5]. This is important in selection as well as training evaluation processes. Identification of younger players who have good physical potentials for basketball game reduces the probability of false selection, while early detection of deficits in the main physical abilities indicates that the training could be adjusted and may reduce the risk of unwanted injuries.

Managing the selection and training process depends on the adequacy of the assessment system in collecting information on athlete’s or a team’s training level in order to provide a precise evaluation of training level [6,7,8]. Furthermore, the usability of results obtained by the assessment depends on the specificity and sensitivity of the applied tests. The more specific the test is with regard to sport, the representation of competitive readiness is more valid [1,8,9]. If the correct data is collected from athletes, the coach can follow the trend in core physical abilities of basketball players through the age categories, and he can timely correct the training program to attain the short, medium, and long-term goals.

The available bibliography reveals the lack of design and use of specific tests to assess the physical attributes of the young basketball players, especially according to age categories and playing positions [1,8]. Growth and maturation affect physical abilities and physical performance [10,11,12], while different basketball positions present different demands and require specific physical attributes [13,14,15]. According to the results of a previous systematic review [1], the least common evaluated capacities in basketball players in literature are speed and agility. Tests of a generic nature have more frequently been used for assessing physical fitness in basketball players, e.g., aerobic and anaerobic capacity or jump performance [1,3,8,9,13,14,15,16]. Besides, only a few pieces of research have dealt with specific tests while dribbling the ball in basketball [1,8]. Consequently, talent identification, selection, and evaluation of training processes are very important parts of the systematic approach to the consistent competitive success of basketball team.

Considering the aforementioned factors, this study aimed to determine the hierarchical structure of physical characteristics in elite young (i.e., U17-U19) basketball players according to playing positions. In addition, their predictive value of physical characteristics was determined for the evaluation of players’ physical preparedness. It was, firstly, hypothesized that significant hierarchical structure of physical abilities will be determined. Secondly, it was hypothesized that the highest ranked variables from the hierarchical structure could be the best predictors of players’ physical performance.

## 2. Materials and Methods

### 2.1. Participants

The sample consisted of 60 male basketball players from the U19 and U17 Serbian national team. In order to obtain the most informative indicators to improve the technological process of managing, we recruited relatively large samples of participants to secure a sufficient statistical power. Besides, we selected a group of elite basketball players who won eight international medals in a period of four years during the biggest World and European competitions. They were allocated into three groups according to playing positions, as follows: Guards (*n* = 28, i.e., point guard and shooting guard), forwards (*n* = 22, i.e., small forward and power forward) and centers (*n* = 10). Basic characteristics of the tested sample were: age = 17.36 ± 1.04 years, 18.00 ± 1.00 years, 17.60 ± 1.43 years; body height = 192.80 ± 4.49 cm, 201.48 ± 3.14 cm, 207.20 ± 3.29 cm; body mass = 79.83 ± 6.94 kg, 90.93 ± 9.85 kg, 104.00 ± 9.64 kg; and training experience = 9.38 ± 2.10 years, 9.93 ± 2.28 years, 9.20 ± 1.62 years for guards, forwards and centers, respectively. All participants (athletes, coaches, and parents) were informed that their data may be used anonymously for scientific purposes and they were informed about the potential risks and discomforts associated with the investigation, and measurements were conducted out with their parental consent in line with the Helsinki Declaration. The Institutional Ethics Committee approved the research.

### 2.2. Measurement Procedure

All the tests were performed by the Serbian Institute of Sport and Sports Medicine at the beginning of the main pre-competitive mesocycle. Players were requested to refrain from strenuous exercise for at least 48 h, and from eating 2 h before testing. The testing session was carried out during morning hours between 10:00 and 12:00 a.m.

Before tests, players had performed a standardised warm-up, consisting of 5 min jogging, 5 min dynamic stretching, and 5 min of short acceleration-decelerations, gradual building of running velocity, submaximal jumping, and agility exercises. For the last five minutes of warm-up, players performed tests with submaximal intensity to potentiate specific muscles and joints. It is of note that the Serbian Institute of Sport and Sports Medicine asses the best Serbian athletes (i.e., members of the national teams) on a regular basis so the used tests were familiar to athletes. The assessment protocol for basketball athletes consists of sprint tests (with and without the ball), change of direction speed tests (with and without the ball), and vertical jump tests. Straight run speed, change of direction speed, and vertical jump heights were measured using Infrared timing gates and contact mat (Fusion sport, SmartJump and SmartSpeed, Grabba International Pty Ltd., Australia). The time of the run dribble was measured in seconds, with an accuracy of ±0.01 s. Jump tests are characterized by a very good test–retest reliability (in general Intraclass correlation coefficients are higher than 0.90) [13,16,17].

#### 2.2.1. Sprint Tests

A 20 m sprint was performed from the standing position with the front foot placed on the line 30 cm behind the photocells. Times were recorded by infrared timing gates placed at the start, at 5 m (first-step quickness [Q5m]), 15 m (acceleration [A15m]), and finish line (Sprint 20 m [S20m]). Players performed the 20 m sprint two times without the ball and two times while dribbling the ball (S20m_D_). The best time obtained from the trial was used for statistical analysis [8,13,18].

#### 2.2.2. Change of Direction Speed with and without the Ball

The following five tests were used to assess change-of-direction speed: *t*-test (T_TEST_), Slalom, Control dribble test (COND), Defensive movements test (DM), Change of direction speed test [2,3,8,16,18]. For the purpose of this study, we applied the standardized procedures used in the previous study [8].

T_TEST_ requires the athlete to move in a T-shaped pattern. According to earlier described procedures [8,13,16,18], the photocells were placed at the starting line and in line with central cone positioned 9 m away from starting position. The athletes started from the standing position, and ran forward 9 m as fast as possible. Then, they shuffled 4.5 m laterally to the left without crossing their feet to another cone. After touching this cone, they shuffled laterally 9 m to the right to a third cone, touched it, side shuffled back to the middle cone, and ran backward to where they started.

In case of Slalom (Slalom), and Slalom while dribbling the ball (Slalom_D_), each participant started the test with his feet behind the baseline of the basketball court. Subjects were required to run (dribble), as fast as possible up and down the course around the three cones placed linearly with 2.6 m distance. They performed two trials with and without the ball and the fasters ones were used for the analysis [8].

**Figure 1:**
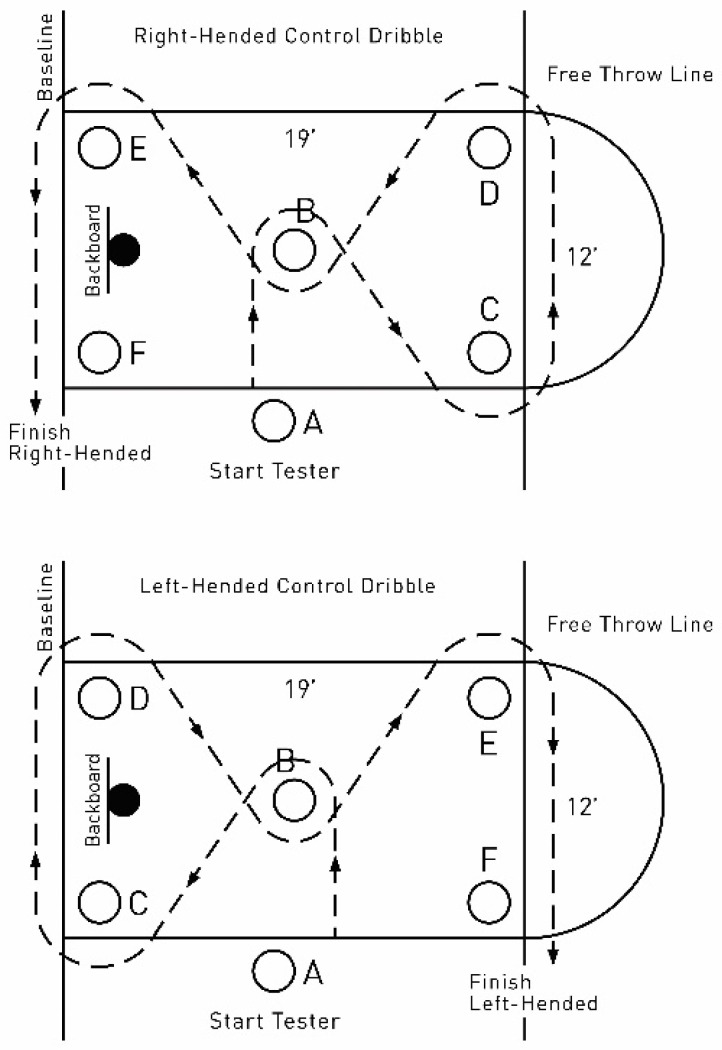
Schematic illustration of the Control of dribbling test. A, B, C, D, E, F—cones; dotted arrow—direction of dribbling.

The COND test was performed at the 5.8 m × 3.6 m rectangle polygon marked by six cones positioned as follows: two at both ends of the free-throw lane, two at the baseline aligned with those at the free-throw line, one in the middle of the rectangle, and one that marked the starting point [8]. The athletes were required to navigate dribbling through a course as fast as possible. The athletes started with their non-dominant hand on the non-dominant side of cone A. They dribbled with non-dominant hand to the non-dominant side of cone B, and then proceed to cone C and cone D, dribbling with the dominant hand. The course continued with the non-dominant hand to cone E and then with the dominant hand to cone F where the test was completed (Figure 1). Three trials were completed for the test. The first was a practice trial and the sum of the second (starting with non-dominant hand) and third trials (starting with dominant hand) was retained for analysis.

**Figure 2:**
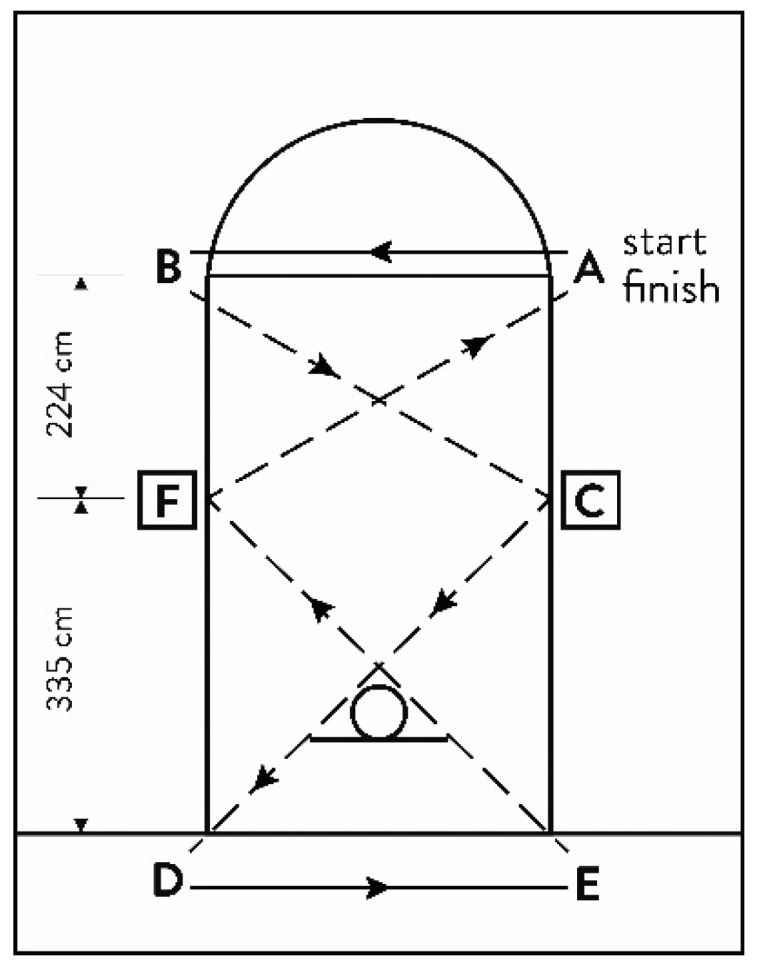
Schematic illustration of the Defensive movements test. A, B, C, D, E, F—cones; dotted arrow—direction of movements.

The DM was used to evaluate the performance of defensive movements. It was performed at the same rectangle polygon as COND, but two cons were positioned at the halfway point of the longer edges of the rectangle. This test was carried following the procedure described in a previous study [8]. The player was required to shuffle laterally without crossing the feet in a sequence of seven changes of direction. Whenever the players changed direction, they were required to touch the floor and execute a drop-step (changing direction by moving the trailing foot in the sliding motion to the new direction (Figure 2). The fastest of the two trials was recorded for the analysis.

A change of direction test (COD) consists of a sprint with several changes of direction. The athletes started in the triple-threat position behind the baseline of the basketball court. Players were required to run (dribble) and to change direction as fast as possible to two different lines, namely, the near free-throw line (5.8 m) and the half-court line (14 m). The athletes sprinted to the free-throw line first and back to the baseline, then to the half-court line and back to the baseline, and finally to the free-throw line again and back to the baseline. Before every change of direction, they were required to step on the line with one foot. After changing direction, they were required to change the dribbling hand. Each athlete was allowed two trials with and without the ball and the fastest one was retained for analysis. Two players performed the test at the same time to encourage maximal effort [8].

#### 2.2.3. Vertical Jumps

The following four types of vertical jump were performed: Sargent jump (SGJ), Squat Jump (SJ), Countermovement jump with arm swing (CMJ_AS_) and Countermovement jump (CMJ). In case of SGJ, the athlete chalked the end of his/her fingertips, stood sideways onto the wall, kept both feet on the ground, reached up as high as possible with one hand and marked the wall with the tips of the fingers (M1). From a static position, they jumped as high as possible and marked the wall with the chalk on their fingers (M2). The distance between M1 and M2 was used to calculate jump height. The athlete repeated the test 2 times [2]. SJ and CMJ vertical jump height were performed according to well-established procedures [13,16,17,18]. In short, SJ was performed from the 90-degree semi-squat position using only the maximal contraction of lower limbs, while CMJ was performed utilizing the energy from the stretch–shortening cycle. In SJ and CMJ, hands were kept at the hips for the entire movement to eliminate any influence of the arm. A CMJ_AS_ was performed the same way as CMJ but players were allowed to swing with their hands upward. Two maximal jumps were performed, and the highest result was registered as the final result.

### 2.3. Statistical Procedures

The mean and standard deviation values for each test were calculated for each subgroup (guards, forwards, and centers). For all the tests involving several trials, test–retest reliability was assessed using intraclass correlation coefficients (ICC). For defining the structure, i.e., real qualitative relationships between variables, the principal component analysis (PCA) was used. A multivariate assessment of the adequacy of the raw data was carried out using the Kaiser-Meyer-Olkin (KMO) measure of sampling adequacy and Bartlett’s Tests of sphericity (*p* < 0.001), for which statistical significance was expressed in terms of a chi-square (χ^2^). Eigenvalues > 1 were considered for the extraction of principal components. A Direct Oblimin rotation method was performed in order to identify the high correlation of components and guarantee that each principal component offered different information [19]. A criterion variable from factor analysis was used as a representation of the player’s multidimensional physical performance index (PP_INDEX_) according to playing position so each player could be compared against the criterion value for their playing position [20]. Multiple regression analysis with the PP_INDEX_ as the criterion variable and the performance test variables as predictor variables determined the unique evaluation of specific preparedness of basketball players according to playing position [20]. Statistical significance for all analyses was defined as *p* < 0.001. All statistical operations were carried out by applying the Microsoft^®^ Office Excel 2010 and the SPSS for Windows, Release 20.0 (Copyright © SPSS Inc., Chicago, IL, USA, 1989–2002).

## 3. Results

Results for the descriptive statistics (Mean and Standard deviation) of the observed characteristics with regard to different playing position and Intraclass correlation coefficients (ICCs) for relative test–retest reliability are shown in Table 1. It can be observed that, in terms of positions, forwards were faster than guards and centers in Q5m and Q5m_D_, while guards were faster than forwards and centers in the majority of change-of-direction speed and sprint tests. In addition, guards achieved a greater jump height compared with forwards and centers. The average inter-item correlation in all variables described mutual correlation within a correlation matrix at a statistically significant level at *p* < 0.001 (Bartlett’s test of Sphericity) and ranged between 0.689 for A15m_D_ and 0.992 for CMJ, indicating a good reliability.

The KMO showed a high statistical significance of multivariate adequacy of the given variables at the level of 0.561 (χ^2^ = 848. 338, *p* < 0.001) for guards, at the level of 0.677 (χ^2^ = 689.135, *p* = 0.001) for forwards, and at the level of 0.558 (χ^2^ = 744.770, *p* = 0.001). For all playing positions, the factor analysis extracted three significant factors (Table 2), which cumulatively explained 76.867, 88.123 and 87.633% of variance in guards, forwards, and centers, respectively.

Table 3 shows the structure matrix with the variable saturation for each playing position. Measured physical characteristics provide a similar factor structure for each position, with a lateral change of direction speed being highly ranked in guards, jumping ability in forwards, and change of direction speed between baseline and free-throw line in centers. The second factor included straight-run speed measures with and without the ball for all three positions. The third factor included a jumping performance in guards, change of direction speed while dribbling the ball, defensive movement in forwards, and jumping performance in centers (with emphasis on jumps with arm swings). This suggests that the measured characteristics with regard to different playing positions have different structures in the function of isolated factors, which may be attributed to their adaptation to specific training process.

The results of the defined regression analysis have shown high predictive potential for PP_INDEX_ of guards (AdjR^2^ = 0.893, F = 165.597, *p* < 0.001, Standard Error of the Estimate = 0.33), forwards (AdjR^2^ = 0.896, F = 170.577, *p* < 0.001, Standard Error of the Estimate = 0.31), and centers (AdjR^2^ = 0.875, F = 138.412, *p* < 0.001, Standard Error of the Estimate = 0.34). The final mathematical models for evaluation of PP_INDEX_ of guards, forwards, and centers is as follows:

PP_INDEX_ of guards = −6.860 + (0.932 × *T* test) − (1.656 × Acceleration 15 m) − (0.020 × Countermovement jump),

PP_INDEX_ of forwards = −3.436 − (0.046 × Countermovement jump with arm swing) − (1.295 × Acceleration 15 m) + (0.582 × Control of dribbling),

PP_INDEX_ of centers = −4.126 + (0.604 × Control of dribbling) − (1.315 × Acceleration 15 m) − (0.037 × Sargent jump).

In this manner, by a very simple mathematical model, coaches could be provided with a tool for the evaluation of players’ physical preparedness according to position, in terms of a deterministic, fully controlled system.

**Figure 3:**
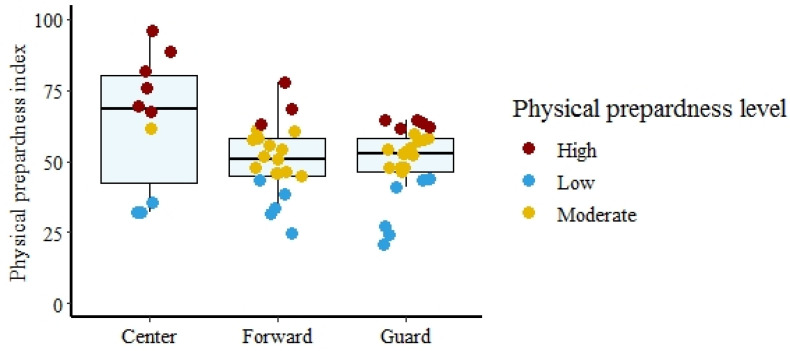
Positioning of players according to playing position from the aspect of specific preparation level.

The regression analysis further reduced the multidimensionality of players’ physical preparedness to the most essential components that predict the PP_INDEX_ of young players with high precision. The best predictors in guards included T_TEST_, A15m, CMJ. The best predictors in forwards were CMJ_AS_, A15m, COND. The best predictors in centers were COND, A15m, and SGJ. Thus, the highest-ranked variables in each factor were the best predictors of PP_INDEX_ for the corresponding positions. The regression model allows for the qualitative and quantitative evaluation of players on the three investigated positions (See Figure 3).

## 4. Discussion

This study investigated the hierarchical structure of physical characteristics in elite young basketball players and evaluated their predictive value in the evaluation of players’ physical preparedness. The main findings showed that specific change of direction speed performance was the highest-ranked characteristic in guards, specific jumping performance was the highest-ranked characteristic in forwards, while the control of specific movements while dribbling the ball was the highest-ranked characteristic in centers. Moreover, a significant prediction model for the evaluation of physical preparedness was defined for each playing position. These findings are of high importance as they provide a screening tool for selection and training evaluation processes.

Considering the structure of the basketball game, players are required to perform numerous technical–tactical elements characterized by agile movements in space in a planned manner or as a response to the opponent’s actions [21]. Shooting guards within their roles and duties perform a higher number of lateral shuffles, forward and backward sprints on relatively bigger area than centers or small forwards. This could be attributed to the role in the game that guards have, such as losing the defender further from the basket by quick directional changes with and without the ball and quick return to defend the basket. Therefore, running speed, agility, and rapid recovery are critical fitness components, particularly for this position [9,13,14,15].

Forwards, on the other hand, typically perform a high number of jumps whether offensively to score the basket or defensively when rebounding, which is also emphasized in the training process. Thereby, jumping characteristics are of high importance for this position. Basketball players typically perform 40–50 jumps per game, generating force rapidly to perform various tasks such as rebounding, blocking opponent shot attempts, and creating elevation for a jump shot [2]. The movement structure of CMJ and CMJ_AS_ corresponds to the bilateral vertical jumps that players most often perform when they are shooting from the distance to advance their ball release height and when they are trying to block the opponent. In addition, forwards are also responsible for the quick return to defence and to defend the space by quick lateral shuffles.

Centers are usually referred to as “frontcourt”, often acting as their team’s primary below-the-basket rebounders and shot blockers. They also receive passes to take inside shots for which they must control their body, opponent, and the ball. Therefore, it is not surprising that centers, as major players on the team, require a high level of control of the specific movement with the ball to maintain their body position when battling with the opponents for important positions under the basket. However, considering the game rules that do not allow staying below the basket longer than 5 s, center is required to move constantly in a square-shaped space from the baseline to the free-throw line. They need to be agile compared to other centers so they could position themselves in a good position repeatedly.

The second factor consisting of acceleration and sprint for all positions indicated the importance of these characteristics for basketball players. Short sprints represent a multidimensional movement skill that requires an explosive concentric and SSC force production of a number of lower-limb muscles [22]. During a game, the players are rarely in a situation where they have to sprint across the whole court. Therefore, sprint tests over shorter distances and acceleration are more appropriate to administer to basketball players [3,8,22]. Indeed, to a large degree (certainly more than power and agility) speed is genetically predetermined, thereby fast players are selected rather than “made fast”, especially considering the sample of this study that consisted of elite players for their age category [23]. This does not reduce the importance of this factor, but additionally suggests that the applied strength and conditioning training could include strength and power exercises that may additionally improve running speed and acceleration or reduce the risk of injury caused by these activities [24,25,26]. It is interesting to mention that center were slightly faster than forwards in the A15 and A15_D_ tests (Table 1). However, if an index of technical efficiency is calculated (the ratio between the A15 and A15_D_ tests), it may be concluded that forwards are still more efficient than centers. Although there are no data in the available literature on the 15 m acceleration test in basketball, results of some previous research showed no significant difference between playing position in the 20 m sprint [9,13]. Even more, these authors suggested that despite their size and weight, centers are as fast as smaller players. Besides that, significant effect of playing position on sprint performance increase in shorter (10 m and shorter) and longer (20 m and longer) distance [13,14] which strongly support our findings.

The last factor is vertical jump performances in guards and centers, showing that these characteristics, although not dominant, are a very important pillar of a basketball player’s physical preparedness. The most representative variable in the third factor was the countermovement jump for guards and Sargent jump for centers. The obtained differences in hierarchy of this factor correspond to differences in how guards and centers perform jumps in the game. Guards are typically jumping free from the opposing player (i.e., no contact with opposing player) and from previously performed movement, while centers are typically jumping from the spot, while in contact with the opposing player with one hand and reaching high with the other to block, rebound or score. Unlike guards and centers, the third factor extracted change of direction speed and speed variables, whereby most representatives were the control of dribbling. This is not surprising, given that forwards often perform dribble penetration to advance to the basket [9,13,14,15].

There is a scarcity of studies that address the specific characteristics of physical fitness in basketball players, even though the battery of basic performance tests are widely used. The reason for that could lie in a fact that possibility of providing a sample of the best selected players for the age category is low. Research dealing with the hierarchical structure and equation of specification in relation to specific performance tests in basketball is practically non-existent in the available literature. The lack of reference to these problems has certainly reduced the possibility to compare our findings with other studies. Data on the defined latent structure of standard indicators of situational efficiency in the game of basketball [27] or in relation to the tests of generic nature [28] can be found in the available literature. The results of that research have shown that the highest total variance in 13 male and 13 female semiprofessional basketball players was represented by aerobic capacity and in-game physical conditioning [28]. In addition, as one of the main limitations of this study, the authors mentioned the need for the inclusion of specific basketball-field tests (e.g., agility with and without the ball, anaerobic capacity) to evaluate the physical performance of basketball players [28]. In relation to the research that used similar methodology [20], the results suggest that it is possible to create sport- position-specific prediction model for evaluation physical preparedness.

### Limitations

However, some limitations should be acknowledged when interpreting the results of this research. An apparent limitation of this study is the results may not be generalized to other age groups or females. In order to apply the obtained results in general, it is necessary to conduct extensive research that includes the examination of physical ability on a large sample of basketball players, of different ages, competitive level and for both genders. Another limitation originates from the cross-sectional design that does not allow for the identification of the effects of physical activity from the initial selection of the subjects.

## 5. Conclusions

The results obtained in this research show that the measured characteristics with regard to different playing positions have different structures in the function of isolated factors under the influence of different mechanisms with regard to the training process. As a factor analysis has a primarily discriminatory character, the first factor with observed variables where the basketball players differ most is the most important one. Specific change direction agility abilities, i.e., specific locomotion on the court is the most important element within guard position in elite youth basketball players. Specific jumping ability is the most important element within the forward position. Control of specific movement with the ball is the most important element within center position.

### Practical Applications

With the multiple regression analysis, the influence of the selected variables on the physical performance index (PP_INDEX_) was obtained, and the equation of specific basketball preparedness according to playing position. This index represents the position of the participant on a hypothetical scale with a minimum of 0 and a maximum of 100 points. In this manner, it is possible to obtain relevant data in relation to physical ability characteristics and, indirectly, to obtain the performance potential of a given athlete. Thus, a useful means for the level of physical fitness determination of youth basketball players has been obtained, as well as a comprehensive reference model for use in selection procedures, screening candidates, or to monitor the efficacy of training regimes.

## Figures and Tables

**Table 1:** Descriptive statistics and Intraclass correlation coefficients.

	Guard	Forward	Center	Test–Retest Reliability	
	Mean ± SD	Mean ± SD	Mean ± SD	Average Int-Item Correlation	Bartlett’s Test of Sphericity
Q5m (s)	1.817 ± 0.413	1.606 ± 0.522	1.820 ± 0.547	0.977	F = 42.775 *
Q5m_D_ (s)	1.883 ± 0.426	1.721 ± 0.647	1.905 ± 0.587	0.732	F = 3.735 *
A15m (s)	1.185 ± 0.378	1.445 ± 0.520	1.372 ± 0.489	0.814	F = 5.384 *
A15m_D_ (s)	1.212 ± 0.396	1.494 ± 0.573	1.437 ± 0.501	0.689	F = 3.218 *
S20m (s)	3.002 ± 0.117	3.052 ± 0.180	3.194 ± 0.128	0.967	F = 30.166 *
S20m_D_ (s)	3.096 ± 0.154	3.215 ± 0.292	3.344 ± 0.162	0.866	F = 7.488 *
T_TEST_ (s)	10.321 ± 0.402	10.481 ± 0.711	11.282 ± 0.695	0.970	F = 33.330 *
DM (s)	18.038 ± 0.860	18.351 ± 1.252	19.601 ± 1.377	0.960	F = 25.079 *
Slalom (s)	4.117 ± 0.178	4.199 ± 0.253	4.443 ± 0.213	0.956	F = 22.797 *
Slalom_D_ (s)	4.214 ± 0.180	4.349 ± 0.272	4.629 ± 0.292	0.959	F = 24.441 *
COD (s)	11.922 ± 0.463	12.114 ± 0.787	12.582 ± 0.905	0.927	F = 13.773 *
COD_D_ (s)	12.482 ± 0.404	12.713 ± 0.825	13.036 ± 0.653	0.953	F = 21.394 *
COND(s)	12.852 ± 0.840	13.144 ± 1.144	13.643 ± 1.180	/	/
CMJ (cm)	41.16 ± 6.20	39.15 ± 5.93	35.79 ± 4.33	0.992	F = 119.140 *
CMJ_AS_ (cm)	48.77 ± 6.30	47.62 ± 6.76	43.67 ± 5.60	0.984	F = 63.837 *
SJ (cm)	34.59 ± 5.77	33.49 ± 5.70	30.28 ± 4.69	0.974	F = 38.620 *
SGJ (cm)	49.34 ± 6.61	48.05 ± 7.66	43.02 ± 3.72	0.929	F = 14.044 *

* T_TEST_: *t* test total time; DM: Defensive movements; S20m_D_: Sprint with dribbling 20 m; COD_D_: Change of direction with dribbling; Slalom: Slalom; Slalom_D_: Slalom with dribbling; A15m_D_: Acceleration with dribbling 15 m; COD: Change of direction; COND: Control of dribbling; S20m: Sprint 20 m; A15m: Acceleration 15 m; Q5m_D_: Quickness with dribbling 5 m; Q5m: Quickness 5 m; CMJ: Countermovement jump without arm swing; SJ: Squat jump; CMJ_AS_: Countermovement jump with arm swing; SGJ: Sargent jump; * *p* values: *p* = 0.000.

**Table 2:** Saturated factors with the structure indicators of the explained variance.

Factor	Extraction Sums of Squared Loadings								
	Total			% of Variance			Cumulative %		
	Guard	Forward	Center	Guard	Forward	Center	Guard	Forward	Center
1	6.719	9.997	11.333	39.523	58.804	66.664	39.523	58.804	66.664
2	3.644	3.899	2.465	21.435	22.936	14.499	60.958	81.739	81.162
3	2.705	1.085	1.100	15.909	6.384	6.471	76.867	88.123	87.633

**Table 3:** Factor analysis structure matrix for each playing position.

Factor	Guard		Forward		Center	
	Variables	Value	Variables	Value	Variables	Value
1st factor	T_TEST_	0.804	CMJ_AS_	−0.966	COND	0.962
	DM	0.783	CMJ	−0.957	COD_D_	0.940
	S20m_D_	0.762	S20	0.934	COD	0.916
	Slalom	0.759	T_TEST_	0.918	Slalom	0.891
	Slalom_D_	0.754	SJ	−0.912	T_TEST_	0.887
	COD	0.749	SGJ	−0.897	Slalom_D_	0.848
	COD_D_	0.749	Slalom	0.878	DM	0.834
	COND	0.720	Slalom_D_	0.834	S20m	0.821
	S20m	0.624	COD_D_	0.823	S20m_D_	0.818
2nd factor	A15m	−0.984	A15m	−0.983	A15m	0.993
	Q5m_D_	0.984	A15m_D_	−0.977	A15m_D_	0.990
	Q5m	0.980	Q5m	0.967	Q5m	−0.980
	A15m_D_	−0.973	Q5m_D_	0.959	Q5m_D_	−0.969
3rd factor	CMJ	0.974	COND	0.869	SGJ	0.949
	CMJ_AS_	0.944	DM	0.846	CMJ_AS_	0.943
	SJ	0.886	COD	0.828	CMJ	0.871
	SGJ	0.813	S20m_D_	0.827	SJ	0.860

T_TEST_: *t*-test total time; DM: Defensive movements; S20m_D_: Sprint with dribbling 20 m; COD_D_: Change of direction with dribbling; Slalom: Slalom; Slalom_D_: Slalom with dribbling; A15m_D_: Acceleration with dribbling 15 m; COD: Change of direction; COND: Control of dribbling; S20m: Sprint 20 m; A15m: Acceleration 15 m; Q5m_D_: Quickness with dribbling 5 m; Q5m: Quickness 5 m; CMJ: Countermovement jump without arm swing; SJ: Squat jump; CMJ_AS_: Countermovement jump with arm swing; SGJ: Sargent jump.

## Data Availability

The data presented in this study are available on request from the corresponding author.
